# Dietary Fibre Intake and Risks of Cancers of the Colon and Rectum in the European Prospective Investigation into Cancer and Nutrition (EPIC)

**DOI:** 10.1371/journal.pone.0039361

**Published:** 2012-06-22

**Authors:** Neil Murphy, Teresa Norat, Pietro Ferrari, Mazda Jenab, Bas Bueno-de-Mesquita, Guri Skeie, Christina C. Dahm, Kim Overvad, Anja Olsen, Anne Tjønneland, Françoise Clavel-Chapelon, Marie Christine Boutron-Ruault, Antoine Racine, Rudolf Kaaks, Birgit Teucher, Heiner Boeing, Manuela M. Bergmann, Antonia Trichopoulou, Dimitrios Trichopoulos, Pagona Lagiou, Domenico Palli, Valeria Pala, Salvatore Panico, Rosario Tumino, Paolo Vineis, Peter Siersema, Franzel van Duijnhoven, Petra H. M. Peeters, Anette Hjartaker, Dagrun Engeset, Carlos A. González, Maria-José Sánchez, Miren Dorronsoro, Carmen Navarro, Eva Ardanaz, José R. Quirós, Emily Sonestedt, Ulrika Ericson, Lena Nilsson, Richard Palmqvist, Kay-Tee Khaw, Nick Wareham, Timothy J. Key, Francesca L. Crowe, Veronika Fedirko, Petra A. Wark, Shu-Chun Chuang, Elio Riboli

**Affiliations:** 1 Department of Epidemiology and Biostatistics, School of Public Health, Imperial College London, London, United Kingdom; 2 International Agency for Research on Cancer (IARC-WHO), Lyon, France; 3 The National Institute for Public Health and the Environment (RIVM), Bilthoven, The Netherlands; 4 Department of Gastroenterology and Hepatology, University Medical Centre, Utrecht, The Netherlands; 5 Institute of Community Medicine, University of Tromsø, Tromsø, Norway; 6 Department of Cardiology, Aarhus University Hospital, Aalborg, Denmark and Department of Epidemiology, School of Public Health, Aarhus University, Aarhus, Denmark; 7 Department of Epidemiology, School of Public Health, Aarhus University, Aarhus, Denmark; 8 Danish Cancer Society, Institute of Cancer Epidemiology, Copenhagen, Denmark; 9 Inserm, Centre for Research in Epidemiology and Population Health, U1018, Institut Gustave Roussy, Villejuif, France; 10 Paris South University, UMRS 1018, Villejuif, France; 11 German Cancer Research Centre, Division of Cancer Epidemiology, Heidelberg, Germany; 12 Department of Epidemiology, German Institute of Human Nutrition, Potsdam-Rehbrücke, Germany; 13 WHO Collaborating Center for Food and Nutrition Policies, Department of Hygiene, Epidemiology and Medical Statistics, University of Athens Medical School, Athens, Greece; 14 Hellenic Health Foundation, Athens, Greece; 15 Department of Epidemiology, Harvard School of Public Health, Boston, Massachusetts, Untied States of America; 16 Bureau of Epidemiologic Research, Academy of Athens, Athens, Greece; 17 Molecular and Nutritional Epidemiology Unit, Cancer Research and Prevention Institute – ISPO, Florence, Italy; 18 Nutritional Epidemiology Unit, Fondazione IRCCS Istituto Nazionale Tumori, Milan, Italy; 19 Department of Clinical and Experimental Medicine, Federico II University, Naples, Italy; 20 Cancer Registry and Histopathology Unit, “Civile - M.P. Arezzo” Hospital, Ragusa, Italy; 21 HuGeF Foundation, Torino, Italy; 22 Division of Human Nutrition, Wageningen University, Wageningen, The Netherlands; 23 Julius Centre, University Medical Centre Utrecht, Utrecht, The Netherlands; 24 Department of Nutrition, Institute of Basic Medical Sciences, University of Oslo, Oslo, Norway; 25 Unit of Nutrition, Environment and Cancer, Cancer Epidemiology Research Program, Catalan Institute of Oncology, Barcelona, Spain; 26 Andalusian School of Public Health, Granada, Spain; 27 CIBER Epidemiology and Public Health CIBERESP, Spain; 28 Public Health Division of Gipuzkoa., Basque Regional Health Department and Ciberesp-Biodonostia, San Sebastian, Spain; 29 Department of Epidemiology, Murcia Regional Health Authority, Murcia, Spain; 30 Navarre Public Health Institute, Pamplona, Spain; 31 Public Health and Health Planning Directorate, Asturias, Spain; 32 Department of Clinical Sciences in Malmö, Lund University, Sweden; 33 Department of Public Health and Clinical Medicine, Medicine, Umeå University, Umeå, Sweden; 34 Medical Bioscience, Umeå University, Umeå, Sweden; 35 University of Cambridge, Cambridge, United Kingdom; 36 MRC Epidemiology Unit, Cambridge, United Kingdom; 37 Cancer Epidemiology Unit, Nuffield Department of Clinical Medicine University of Oxford, Oxford, United Kingdom; Sookmyung Women's University, Republic of Korea

## Abstract

**Background:**

Earlier analyses within the EPIC study showed that dietary fibre intake was inversely associated with colorectal cancer risk, but results from some large cohort studies do not support this finding. We explored whether the association remained after longer follow-up with a near threefold increase in colorectal cancer cases, and if the association varied by gender and tumour location.

**Methodology/Principal Findings:**

After a mean follow-up of 11.0 years, 4,517 incident cases of colorectal cancer were documented. Total, cereal, fruit, and vegetable fibre intakes were estimated from dietary questionnaires at baseline. Hazard ratios (HRs) and 95% confidence intervals (CIs) were estimated using Cox proportional hazards models stratified by age, sex, and centre, and adjusted for total energy intake, body mass index, physical activity, smoking, education, menopausal status, hormone replacement therapy, oral contraceptive use, and intakes of alcohol, folate, red and processed meats, and calcium. After multivariable adjustments, total dietary fibre was inversely associated with colorectal cancer (HR per 10 g/day increase in fibre 0.87, 95% CI: 0.79–0.96). Similar linear associations were observed for colon and rectal cancers. The association between total dietary fibre and risk of colorectal cancer risk did not differ by age, sex, or anthropometric, lifestyle, and dietary variables. Fibre from cereals and fibre from fruit and vegetables were similarly associated with colon cancer; but for rectal cancer, the inverse association was only evident for fibre from cereals.

**Conclusions/Significance:**

Our results strengthen the evidence for the role of high dietary fibre intake in colorectal cancer prevention.

## Introduction

A possible protective association between dietary fibre intake and colorectal cancer was first proposed by Burkitt in 1971. [1] Putative anti-carcinogenic mechanisms of dietary fibre within the bowel include: the formation of short-chain fatty acids from fermentation by colonic bacteria; the reduction of secondary bile acid production; the reduction in intestinal transit time and increase of faecal bulk; and a reduction in insulin resistance.[2]–[4] Inverse associations between dietary fibre intake and colorectal cancer risk have also been reported in ecological and case-control studies. [5], [6] However, the evidence from prospective studies has been inconsistent,[7]–[15] with the two largest analyses published to date yielding non-significant associations. [13], [14] In both the Pooling Project [13] and NIH (National Institutes of Health)-AARP analyses [14], statistically significant inverse associations in age-adjusted models disappeared after multivariable adjustment. In contrast, inverse associations for colorectal adenoma [8] and colorectal cancer[9]–[12] have been reported in other prospective studies. In the EPIC study after an average 6.2 years of follow-up, and 1,721 colorectal cancer cases, a 21% reduced risk amongst participants in the highest intake quintile was observed when compared against the lowest intake group. [16].

Differing adjustments for colorectal cancer risk factors which may confound the dietary fibre relationship (such as dietary folate) has been proposed as a possible explanation for the variable results observed between studies. [13], [17] This is because high dietary fibre intake is usually correlated with other lifestyle and dietary factors which are also associated with colorectal cancer. The risk of possible residual confounding was acknowledged in the 2007 World Cancer Research Fund and American Institute for Cancer Research (WCRF/AICR) expert report, in which the fibre-colorectal cancer association was classified as “probable” rather than “convincing". However, in a more recent systematic review, in which higher concordance between study results was observed, the WCRF/AICR panel upgraded the fibre-colorectal association to “convincing”. [18] The review concluded that further detailed analyses by colorectal sub-site, and fibre source are warranted. [19] Within that review, EPIC was one of the largest and most influential studies reporting an inverse association. The aims of the present study were to examine whether the previously observed inverse association persisted after longer follow-up (mean 11 years) and an increased number of colorectal cancer cases (from n = 1,721 to n = 4,517); to provide a more precise estimation of the association by cancer sub-site and fibre food source; and to scrutinise the fibre-colorectal cancer relationship further by examining possible interactions by age, sex, and other lifestyle, anthropometric, and dietary variables.

## Materials and Methods

### Outline

EPIC is an ongoing multicentre prospective cohort study designed to investigate the associations between diet, lifestyle, genetic and environmental factors and various types of cancer. A detailed description of the methods has previously been published. [20], [21] In summary, 521,448 participants (∼70% women) mostly aged 35 years or above were recruited between 1992 and 2000. Participants were recruited from 23 study centres in ten European countries: Denmark, France, Germany, Greece, Italy, the Netherlands, Norway, Spain, Sweden, and United Kingdom (UK). Participants were recruited from the general population of their respective countries, with the following exceptions: the French cohort were teacher health insurance programme members; the Italian and Spanish cohort included members of blood donor associations and the general population; the Utrecht (the Netherlands) and Florence (Italy) cohorts contained participants from mammographic screening programs; the Oxford (UK) cohort included a large proportion of vegetarians, vegans, and low meat eaters; finally, only women participated in the cohorts of France, Norway, Utrecht and Naples (Italy). Written informed consent was provided by all study participants. Ethical approval for the EPIC study was provided from the review boards of the International Agency for Research on Cancer (IARC) and local participating centres. Exclusions prior to the onset of the analyses included: participants with prevalent cancer at enrolment (n = 28,283); participants with missing dietary or non-dietary data (n = 6,253); and finally participants in the highest and lowest 1% of the distribution for the ratio between energy intake to estimated energy requirement (n = 9,600). Our study therefore included 477,312 participants (335,062 women and 142,250 men).

### Diet and Lifestyle Questionnaires

Dietary information over the previous 12 months was obtained at study baseline using country/centre specific dietary questionnaires. The relative validity and reproducibility of the questionnaires has previously been published. [22] In Malmö, a dietary questionnaire was combined with a 7-day food registration and interview. In Greece, two Italian centres, and Spain, interviewers administered the dietary questionnaires. In all other centres/countries, the questionnaires were self-administered. In Spain, France, and Ragusa (Italy) questions were structured by meals, while in other countries the structure was by food groups. Also at baseline, standardised computer-based single 24-hour dietary recalls (24-hdr) were collected from 36,900 study participants. This additional dietary assessment was used to calibrate for differences in questionnaires across countries. [23] The estimation of fibre intakes from foods within this population has previously been described. [24] Briefly, the AOAC (Association of Official Agricultural Chemists) gravimetric method [25] was used for all countries, except in the UK and Greece, where the Englyst method [26] was used. To take into account the different analytical methods used, the fibre variable used in this analysis was obtained from the EPIC Nutrient Data Base (ENDB); in which the nutritional composition of foods across the different countries has been standardised. [27].

Lifestyle questionnaires were used to obtain information on education (used as a proxy for socioeconomic status), smoking status and intensity, alcohol consumption, physical activity levels, oral contraceptive use, menopausal status, and menopausal hormone use. Height and weight were measured at the baseline examination in all centres apart from part of Oxford and all of the France and Norway sub-cohorts, where measurements were self reported via the lifestyle questionnaire. [21].

### Ascertainment of Colorectal Cancer Incidence

Population cancer registries were used in Denmark, Italy, the Netherlands, Norway, Spain, Sweden and the United Kingdom to identify incident cancers. In France, Germany and Greece cancer cases were identified through active follow-up, directly through study participants or next of kin, and confirmed by a combination of methods including health insurance records, and cancer and pathology registries. Loss to follow-up across all countries was low (<2%). Complete follow-up censoring dates varied amongst centres, ranging between 2005 and 2010.

Cancer incidence data were coded in accordance with the 10^th^ Revision of the International Classification of Diseases (ICD-10) and the second revision of the International Classification of Disease for Oncology (ICDO-2). Proximal colon cancer included those within the caecum, appendix, ascending colon, hepatic flexure, transverse colon, and splenic flexure (C18.0–18.5). Distal colon cancer included those within the descending (C18.6) and sigmoid (C18.7) colon. Overlapping (C18.0) and unspecified (C18.9) lesions of the colon were grouped among colon cancers only. Cancer of the rectum included cancer occurring at the rectosigmoid junction (C19) and rectum (C20).

### Statistical Analysis

Hazard ratios (HRs) and 95% confidence intervals (CIs) were estimated using Cox proportional hazards models. Age was the primary time variable in all models. Time at entry was age at recruitment. Exit time was age at whichever of the following came first: colorectal cancer diagnosis, death, or the date at which follow-up was considered complete in each centre. To control for differing follow-up procedures, questionnaire design, and other differences across centres, models were stratified by study centre. Models were also stratified by sex and age at recruitment in 1-year categories. Possible non-proportionality was assessed using an analysis of Schoenfeld residuals; [28] with no evidence of non-proportionality being detected. Dietary fibre intakes were modelled using quintiles defined across EPIC participants, and as continuous variables (HRs per 10 g/day intakes of total fibre, cereal fibre, and fruit and vegetable fibre). Trend tests across intake categories were calculated by assigning the median value of each intake quintile and modelling as continuous terms into Cox regression models.

Analyses for colorectal, colon, proximal colon, distal colon, and rectal cancers were conducted for both sexes combined and in men and women separately. All models were adjusted for total energy intake (kcal/day; continuous); body mass index (BMI; kg/m^2^; continuous); physical activity (inactive, moderately inactive, moderately active, active, or missing); smoking status and intensity (never; current, 1–15 cigarettes per day; current, 16–25 cigarettes per day; current, 16+ cigarettes per day; former, quit ≤10 years; former, quit 11–20 years; former, quit 20+ years; current, pipe/cigar/occasional; current/former, missing; or unknown); education level (none/primary school completed, technical/professional school, secondary school, longer education - including university, or unknown); menopausal status (premenopausal, postmenopausal, perimenopausal/unknown menopausal status, or surgical postmenopausal); ever use of oral contraceptives (yes, no, or unknown); ever use of menopausal hormones (yes, no, or unknown); and intakes of alcohol (g/day), folate (µg/day), red and processed meats (g/day), and calcium (mg/day) (all continuous). Possible adjustment for waist circumference instead of BMI was assessed in a subset of the cohort in which measurements were available, but the risk estimates were virtually unchanged; and accordingly, we adjusted for BMI that was available for most participants. We also analysed the association modelling fibre from different food sources (cereal, fruit, and vegetable). These models included the same covariates as detailed above, with additional mutual adjustment for the other fibre sources. Fruit and vegetable fibre intakes were combined to give similar intake categories to the cereal fibre analysis. The relationship between fibre from legumes and colorectal cancer was also assessed, but due to low intakes in the cohort, the results are not shown. In sensitivity analyses, the results were adjusted for total energy using the residual method.

To evaluate whether the total dietary fibre and colorectal cancer relationship varied according to anthropometric, lifestyle, and other dietary variables, we included interaction terms in the model. The statistical significance of the cross-product terms were evaluated using the likelihood ratio test. Interaction terms inputted into the statistical model were fibre intake (continuous; per 10 g/day) with age at recruitment (<55 years, 55 to 65 years, or >65 years); BMI (underweight  =  <18.5 kg/m^2^; normal  = 18.5 to <25 kg m^2^; overweight  = 25.0 to <30 kg/m^2^; or obese  =  ≥30 kg/m^2^); waist circumference, using categories from a previous EPIC analysis on anthropometry and colorectal cancer [29] (women: <70.2, 70.2 to <89, and ≥89 cm; men: <86 cm; 86 to <102; ≥102 cm); smoking status (never, former, or current); physical activity (active, or inactive); alcohol consumption (<30 g/day; and >30 g/day), and intake quartiles of folate, calcium, and red and processed meat.

Cox proportional hazard restricted cubic spline models were used to explore possible deviation from non-linear associations, with five knots specified at the median of each fibre intake quintile. [30] Heterogeneity of associations across cancer sub-sites was assessed by calculating χ^2^ statistics. The heterogeneity across countries was explored by taking a meta-analytic approach. [31] We further combined the country specific risk estimates using a random-effects model.

To improve comparability of data across study centres and to partially correct the relative risk estimates for the measurement error of dietary intakes, a linear regression calibration model was used utilising the 24-hdr taken at baseline from a subset of the cohort (n = 34,436 in this analysis). [32], [33] The 24-hdr were regressed on dietary questionnaire values, with adjustment for the same list of covariates detailed above, and further control for the week day and season of recall measurements. Country and sex-specific calibration models were used to obtain individual calibrated values of dietary exposure for all participants. Cox proportional hazards regression models were then applied using the calibrated values for each participant on a continuous scale. The standard error of the de-attenuated coefficients was corrected through bootstrap sampling. The *P-value* for the trend of the de-attenuated coefficients was calculated by dividing the de-attenuated coefficient by the bootstrap-derived standard error and approximating the standardized normal distribution. [32].

Statistical tests used in the analysis were all two-sided and a *P-value* of <0.05 was considered statistically significant. Analyses were conducted using SAS version 9.1 and Stata version 11.0.

## Results

After a mean follow-up of 11.0±2.8 years, 4,517 colorectal cancer cases were documented amongst the 477,312 participants. Of the 4,517 colorectal cancers, 2,869 were colon (1,266 distal; 1,298 proximal; and 305 overlapping or unspecified), and 1,648 were rectal cancers. The total person-years and distribution of colorectal cancer cases by country are shown in Table 1. The crude colorectal cancer incidence rates for men and women were 12 and 7 cases per 10,000 person-years respectively. The highest total fibre intakes among men were observed in Spain and the highest intakes amongst women were observed in Denmark (Table 1). Men and women from Sweden had the lowest total fibre intakes. Baseline characteristics of study participants by quintile of total fibre intake are shown in Table 2. Men and women in the higher fibre intake groups had a higher proportion of never smokers and physically active participants. Higher fibre intake was also associated with higher average intakes of calcium, and folate; and lower intakes of red and processed meat and alcohol compared to participants with lower fibre intakes.

**Table 1:** Descriptive information of the European Prospective Investigation into Cancer and Nutrition participant countries.

	Number of participants		Total person-years		Number of colorectalcancer cases		Total dietary fibre intake(g/day) *	
Country	Men	Women	Men	Women	Men	Women	Men	Women
France	–	67,385	–	699,360	–	423	–	20.0 (8.7)
Italy	14,029	30,512	158,917	341,489	173	245	25.5 (10.7)	19.6 (8.9)
Spain	15,148	24,854	182,965	299,617	185	144	26.1 (12.7)	20.7 (11.1)
United Kingdom	22,852	52,543	252,096	586,301	324	404	23.3 (10.3)	19.4 (9.3)
The Netherlands	9,639	26,866	115,570	315,683	82	305	25.1 (11.5)	21.3 (8.7)
Greece	10,807	15,225	99,108	148,604	61	44	23.9 (14.8)	17.6 (10.4)
Germany	21,172	27,411	208,509	272,105	265	172	23.0 (10.2)	20.4 (8.8)
Sweden	22,309	26,375	289,623	349,308	339	313	19.3 (8.5)	16.7 (6.9)
Denmark	26,294	28,722	284,721	316,745	475	353	26.0 (11.0)	23.1 (9.8)
Norway	–	35,169	–	342,279	–	210	–	19.1 (8.3)
All EPIC	142,250	335,062	1,591,508	3,671,490	1,904	2,613	23.7 (11.4)	19.8 (9.1)

* Data are mean and (SD) of dietary fibre intake information collected from 24-hour dietary recalls (n = 34,436 participants).

**Table 2:** Characteristics of study participants by categories of dietary fibre intake.

Quintile of dietary fibre intake	Q1		Q2		Q3		Q4		Q5	
**Fibre range (energy adjusted; g/day)**	<17.9		17.9–<21.0		21.0–<23.6		23.6–<27.5		≥27.5	
**Fibre range (actual; g/day)**	<16.4		16.4–<20.1		20.1–<23.6		23.6–<28.5		≥28.5	
**Men**										
***N***	21,675		22,590		25,834		31,664		40,487	
**Colorectal cancer cases**	328		296		392		400		488	
**Age at recruitment (years)** §	51.8	10.1	51.8	10.0	52.1	10.0	52.5	10.1	52.5	10.3
**Body mass index (kg/m^2^)** §	26.4	3.7	26.6	3.6	26.6	3.6	26.7	3.6	26.3	3.7
**Education**										
Longer education inclu. uni. (%)	21.2		23.9		25.7		26.8		31.1	
**Smoking status**										
Never (%)	25.8		30.0		31.7		33.5		39.0	
Current (%)	40.9		33.8		30.7		27.5		21.4	
**Physical activity**										
Active (%)	21.4		22.4		23.5		24.1		27.3	
**Total energy intake (kcal/day)** §	2,366	681	2,423	665	2,443	668	2,434	654	2,386	651
**Red and processed meat intake (g/day)** §	116.2	59.6	108.9	54.5	103.3	53.1	96.7	52.1	81.3	55.1
**Calcium intake (mg/day)** §	1037	384	1029	342	1034	330	1,043	329	1,084	340
**Folate intake (µg/day)** §	249.1	66.3	278.8	64.7	301.2	68.6	326.8	75.1	394.4	125
**Alcohol intake (g/day)** §	30.8	32.6	24.2	24.7	20.9	21.6	18	19.2	13.9	15.7
**Women**										
***N***	73,788		72,873		69,628		63,798		54,975	
**Colorectal cancer cases**	583		543		559		502		426	
**Age at recruitment (years)** §	50.2	9.7	50.7	9.6	51.1	9.5	51.3	9.6	50.8	10.8
**Body mass index (kg/m^2^)** §	24.7	4.4	25	4.5	25.1	4.5	25.1	4.4	24.9	4.4
**Education**										
Longer education inclu. uni. (%)	22.0		22.1		21.9		22.3		25.4	
**Smoking status**										
Never (%)	49.8		55.7		57.1		57.7		59.3	
Current (%)	27.8		20.8		17.9		16.1		12.6	
**Physical activity**										
Active (%)	11.6		12.0		13.2		15.1		19.3	
**Ever use of contraceptive pill**										
Yes %	58.9		56.8		56.5		56.7		57.2	
**Ever use of menopausal hormone therapy**										
Yes (%)	22.5		23.3		24.7		25.9		25.5	
**Menopausal status**										
Postmenopausal (%)	39.9		41.2		44.2		45.9		46.2	
**Total energy intake (kcal/day)** §	1920	561	1961	549	1950	538	1926	522	1890	520
**Red and processed meat intake (g/day)** §	78.4	41.2	71.4	37.5	67.1	37.1	61.5	37.5	46.5	38.2
**Calcium intake (mg/day)** §	979	342	956	296	960	295	978	301	1023	310
**Folate intake (µg/day)** §	244.3	66.3	278	67.9	301	75.8	329	87.8	406	138
**Alcohol intake (g/day)** §	11.6	15.8	8.4	11.5	7.0	9.9	6.2	8.8	5.3	7.8

§ Mean and standard deviation.

* Food and nutrient intakes were sourced from dietary questionnaires and are adjusted for total energy unless stated otherwise.

### Total Dietary Fibre

**Figure 1:**
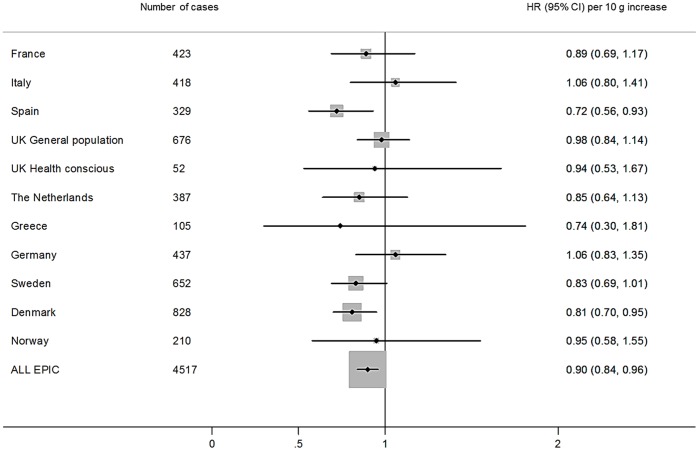
Multivariable hazard ratios and 95% confidence intervals of colorectal cancer risk by country, per 10 g/day increase in total dietary fibre intake. Hazard ratios were estimated by Cox proportional hazard models adjusted for total energy intake (continuous), body mass index (continuous), physical activity index (inactive, moderately inactive, moderately active, active, or missing), smoking status and intensity (never; current, 1–15 cigarettes per day; current, 16–25 cigarettes per day; current, 16+ cigarettes per day; former, quit ≤10 years; former, quit 11–20 years; former, quit 20+ years; current, pipe/cigar/occasional; current/former, missing; unknown), education status (none, primary school completed, technical/professional school, secondary school, longer education including university, or not specified), ever use of contraceptive pill (yes, no, or unknown), ever use of menopausal hormone therapy (yes, no, or unknown), menopausal status (premenopausal, postmenopausal, perimenopausal/unknown menopausal status, or surgical postmenopausal), and intakes of alcohol, folate, red and processed meat, and calcium (all continuous), and stratified by age (1-year categories), sex, and centre. *Uncalibrated model shown.

For colorectal cancer, higher total dietary fibre intake was associated with a statistically significantly reduced risk in the basic model which was adjusted for total energy intake, and stratified by age, sex, and centre (Q5 vs.Q1, HR 0.76, 95% CI: 0.68–0.85, *P-trend* <0.001) (Table 3). This association was attenuated after multivariable adjustment, but a statistically significant 17% lower risk (95% CI: 0.72–0.96; *P*-trend 0.013) remained. The most important confounders influencing this attenuation were alcohol consumption and smoking. Risk estimates were similar when adjusting for energy intake using the residual method (data not shown). In calibrated models, a 13% lower (95% CI: 0.79–0.96) colorectal cancer risk per 10 g/day increase in total fibre intake was yielded. The interaction between sex and total fibre intake was non-significant (*P* for interaction 0.18), therefore combined risk estimates for men and women are shown (separate results for men and women are presented in Tables S1 and S2). No significant interactions for the association of total dietary fibre and colorectal cancer risk were observed for BMI (*P* = 0.75), waist circumference (men *P* = 0.95; women *P* = 0.77), age at recruitment (*P* = 0.83), physical activity (*P* = 0.74), education level attained (*P* = 0.17), smoking (*P* = 0.20), and dietary intakes of alcohol (*P* = 0.20), red and processed meat (*P* = 0.40), folate (*P* = 0.76), and calcium (*P* = 0.20) (data not tabulated). In the restricted cubic spline models, no deviation from linearity for the relationship between total fibre and colorectal cancer was observed (*P* = 0.73) (Figure S1). There was no evidence of heterogeneity by country for total fibre intake and colorectal cancer development (*P* = 0.44) (Figure 1). A similar association for colorectal cancer was found when the country specific risk estimates were pooled using a random-effects model (HR per 10 g/day increase 0.89, 95% CI: 0.83–0.96; data not tabulated).

**Table 3:** Multivariable hazard ratios (95% confidence intervals) of colorectal cancer risk by cohort wide total dietary fibre intake quintiles.

		Quintile of total fibre intake									Uncalibrated	Calibrated
Fibre intake range (g/day)		1	2		3	4		5			HR (95% CI)per 10 g/dayincrease	HR (95% CI)per 10 g/dayincrease
		<16.4	16.4–<20.1		20.1–<23.6	23.6–<28.5		≥28.5		*P*-*trend*		
**Colorectum**												
	*N* cases	931	918		912		914	842				
	Basic†	1.00	0.95 (0.87–1.04)		0.91 (0.83–1.01)		0.88 (0.80–0.98)	0.76 (0.68–0.85)		<0.001		
	Multivariable‡	1.00	0.98 (0.89–1.08)		0.96 (0.86–1.06)		0.94 (0.84–1.05)	0.83 (0.72–0.96)		0.013	0.90 (0.84–0.96)	0.87 (0.79–0.96)
**Colon**												
	*N* cases	611	582		586	571		519				
	Basic†	1.00	0.93 (0.82–1.04)		0.91 (0.80–1.03)	0.86 (0.76–0.98)		0.74 (0.64–0.86)		<0.001		
	Multivariable‡	1.00	0.94 (0.84–1.06)		0.94 (0.83–1.07)	0.91 (0.79–1.04)		0.80 (0.67–0.95)		0.017	0.89 (0.81–0.97)	0.88 (0.80–0.97)
**Colon - proximal**												
	*N* cases	267	250		290	244		247				
	Basic†	1.00	0.93 (0.78–1.11)		1.06 (0.89–1.26)	0.89 (0.73–1.08)		0.86 (0.69–1.07)		0.16		
	Multivariable‡	1.00	0.95 (0.79–1.14)		1.10 (0.91–1.33)	0.93 (0.75–1.15)		0.92 (0.71–1.20)		0.51	0.91 (0.80–1.03)	0.83 (0.75–0.92)
**Colon - distal**												
	*N* cases	286	262	241		263		214				
	Basic†	1.00	0.88 (0.74–1.05)	0.80 (0.66–0.95)		0.84 (0.69–1.01)		0.65 (0.52–0.82)		<0.001		
	Multivariable ‡	1.00	0.90 (0.75–1.07)	0.83 (0.68–1.00)		0.88 (0.71–1.09)		0.70 (0.53–0.92)		0.021	0.88 (0.77–1.00)	0.98 (0.88–1.08)
**Rectum**												
	*N* cases	320	336	326		343		323				
	Basic†	1.00	1.00 (0.85–1.17)	0.92 (0.79–1.09)		0.92 (0.78–1.09)		0.79 (0.65–0.96)	0.012			
	Multivariable‡	1.00	1.04 (0.88–1.22)	0.99 (0.83–1.17)		1.00 (0.83–1.21)		0.90 (0.72–1.14)	0.34		0.92 (0.82–1.02)	0.87 (0.79–0.96)

† Basic model - Cox regression using total energy intake (continuous), and stratified by age (1-year categories), sex, and centre.

‡ Multivariable model - Cox regression using total energy intake (continuous), body mass index (continuous), physical activity index (inactive, moderately inactive, moderately active, active, or missing), smoking status and intensity (never; current, 1–15 cigarettes per day; current, 16–25 cigarettes per day; current, 16+ cigarettes per day; former, quit ≤10 years; former, quit 11–20 years; former, quit 20+ years; current, pipe/cigar/occasional; current/former, missing; unknown), education status (none, primary school completed, technical/professional school, secondary school, longer education including university, or not specified), ever use of contraceptive pill (yes, no, or unknown), ever use of menopausal hormone therapy (yes, no, or unknown), menopausal status (premenopausal, postmenopausal, perimenopausal/unknown menopausal status, or surgical postmenopausal), and intakes of alcohol, folate, red and processed meat, and calcium (all continuous), and stratified by age (1-year categories), sex, and centre.

No significant heterogeneity was seen for the associations between total dietary fibre with colon and rectal cancers (*P* for heterogeneity  = 0.65). In calibrated linear models, the risk estimates were 0.88 (95% CI: 0.80–0.97) and 0.87 (95% CI: 0.79–0.96) for 10 g/day increase of total fibre intake for colon and rectal cancers respectively (Table 3). In categorical analyses, the inverse association with rectal cancer in the basic model disappeared after multivariable adjustment (Q5 vs. Q1, HR 0.90, 95% CI: 0.72–1.14), with alcohol consumption being the main confounder contributing to the attenuation. Within the colon, no strong evidence of a difference in association between cancers located in the distal and proximal regions emerged from our results (*P* for heterogeneity  = 0.72). However, in categorical models, an inverse trend was observed for cancer located in the distal region of the colon, but not for proximal colon cancer; whereas in the calibrated continuous models, a significant inverse association was observed for proximal colon cancers (HR per 10 g/day increase 0.83, 95% CI: 0.75–0.92) but not for distal cancers (HR per 10 g/day increase 0.98, 95% CI: 0.88–1.08).

### Fibre from Different Food Sources

In analyses by fibre food source and colorectal cancer risk - after mutual adjustment for fibre from the other food sources - inverse associations were observed for cereal fibre (HR per 10 g/day 0.89; 95% CI 0.82–0.97), and for fibre from fruits and vegetables combined (HR per 10 g/day 0.91; 95% CI 0.83–1.00) (Table 4). For colon cancer, statistically significant 11% reduced risks were observed for fibre from cereals, and fruits and vegetables combined. When fibre from fruits and vegetables were analysed separately the highest intake category was >6.7 g/day for both sources, and non-significant associations were observed across all colorectal cancer sites (data not shown). For cereal fibre, similar results were observed for colon and rectal cancers. However, fibre from fruits and vegetables combined was not associated with rectal cancer.

**Table 4:** Multivariable hazard ratios (95% confidence intervals) of colorectal cancer risk for fibre source intake quintiles.

		Quintile of fibre intake						
		1	2	3	4	5	*P-trend*	
Cereal fibre (g/day)		<4.64	4.64–<6.72	6.72–<8.97	8.97–<12.3	≥12.3		HR (95% CI) per 10 g/day increase
Colorectum	*N* cases	857	921	972	918	849		
	Basic	1.00	1.06 (0.96–1.16)	1.05 (0.95–1.16)	0.95 (0.85–1.05)	0.83 (0.73–0.93)	<0.001	
	Multivariable	1.00	1.07 (0.97–1.17)	1.07 (0.96–1.18)	0.97 (0.87–1.09)	0.87 (0.77–0.99)	0.003	0.89 (0.82–0.97)
Colon	*N* cases	550	613	608	572	526		
	Basic	1.00	1.03 (0.91–1.16)	1.05 (0.93–1.18)	0.91 (0.80–1.04)	0.86 (0.74–0.99)	0.006	
	Multivariable	1.00	1.03 (0.92–1.17)	1.06 (0.93–1.20)	0.92 (0.81–1.06)	0.88 (0.76–1.03)	0.032	0.89 (0.80–0.99)
Rectum	*N* cases	307	308	364	346	323		
	Basic	1.00	1.11 (0.94–1.31)	1.05 (0.88–1.24)	1.01 (0.85–1.21)	0.78 (0.64–0.95)	0.001	
	Multivariable	1.00	1.13 (0.96–1.34)	1.08 (0.91–1.29)	1.07 (0.89–1.28)	0.86 (0.70–1.06)	0.031	0.89 (0.78–1.01)
**Fruit and vegetable** **fibre (g/day)**		**<5.1**	**5.10–<7.3**	**7.30–<9.62**	**9.62–<12.9**	**≥12.9**		**HR (95% CI) per 10 g/day increase**
Colorectum	*N* cases	969	993	904	849	775		
	Basic	1.00	1.02 (0.93–1.12)	0.94 (0.86–1.04)	0.92 (0.83–1.02)	0.90 (0.80–1.00)	0.016	
	Multivariable	1.00	1.05 (0.95–1.15)	0.98 (0.89–1.09)	0.96 (0.86–1.08)	0.94 (0.82–1.07)	0.19	0.91 (0.83–1.00)
Colon	*N* cases	623	616	555	558	501		
	Basic	1.00	0.95 (0.84–1.06)	0.84 (0.75–0.95)	0.86 (0.76–0.97)	0.81 (0.70–0.93)	0.002	
	Multivariable	1.00	0.97 (0.86–1.09)	0.87 (0.77–0.99)	0.89 (0.77–1.02)	0.83 (0.70–0.98)	0.022	0.89 (0.79–0.99)
Rectum	*N* cases	346	377	349	291	274		
	Basic	1.00	1.15 (0.99–1.34)	1.14 (0.98–1.34)	1.04 (0.88–1.23)	1.08 (0.89–1.29)	0.89	
	Multivariable	1.00	1.19 (1.02–1.39)	1.21 (1.03–1.42)	1.11 (0.92–1.33)	1.17 (0.94–1.45)	0.40	0.96 (0.82–1.12)

† Basic model - Cox regression using total energy intake (continuous), and stratified by age (1-year categories), sex, and centre.

‡ Multivariable model - Cox regression using total energy intake (continuous), body mass index (continuous), physical activity index (inactive, moderately inactive, moderately active, active, or missing), smoking status and intensity (never; current, 1–15 cigarettes per day; current, 16–25 cigarettes per day; current, 16+ cigarettes per day; former, quit ≤10 years; former, quit 11–20 years; former, quit 20+ years; current, pipe/cigar/occasional; current/former, missing; unknown), education status (none, primary school completed, technical/professional school, secondary school, longer education including university, or not specified), ever use of contraceptive pill (yes, no, or unknown), ever use of menopausal hormone therapy (yes, no, or unknown), menopausal status (premenopausal, postmenopausal, perimenopausal/unknown menopausal status, or surgical postmenopausal), and intakes of alcohol, folate, red and processed meat, calcium, and mutual adjustment for fibre from other sources (all continuous), and stratified by age (1-year categories), sex, and centre.

## Discussion

This analysis of the EPIC cohort, after a longer term follow-up of 11 years in which 4,517 cases accrued, further strengthens the evidence that dietary fibre is inversely associated with colorectal cancer risk. The inverse association of total fibre with colorectal cancer risk was of similar magnitude in men and women, and for colon and rectal cancers. No strong evidence of different associations across the distal and proximal regions of the colon was observed. These results support our previous conclusion, of the potential of reducing colorectal cancer incidence by increasing fibre intake from cereal, fruit, and vegetable food sources. [16], [34].

The association of total fibre intake with colorectal cancer has been observed in several prospective studies.[9]–[12] However, null results were reported in the multivariable models of the two largest analyses published to date. [13], [14] In both studies, statistically significant associations in age-adjusted models disappeared after adjustment for other risk factors. Firstly, a Pooling Project analysis including data from 13 cohort studies reported statistically significant inverse associations for colorectal cancer in the age adjusted models (Q5 vs. Q1, RR 0.84, 95% CI: 0.77–0.92), but not after multivariable adjustment (Q5 vs. Q1, RR 0.94, 95% CI: 0.86–1.03). [13] Similarly, in an NIH-AARP analysis the statistically significant inverse association in the age adjusted model (Q5 vs. Q1, HR 0.73, 95% CI: 0.65–0.82) disappeared after multivariable adjustment (Q5 vs. Q1, RR 0.99, 95% CI: 0.85–1.15). [14] Identifying the reasons for these inter-study discrepancies has so far proved elusive. It has been argued that the inverse associations in the EPIC study could have been explained by residual confounding, in particular by lack of adjustment for folate intake. [17] Fibre is especially vulnerable to confounding bias as high intake is usually associated with other practices beneficial to health, such as not smoking, drinking less alcohol, eating less red meat, and being physically active. [17] However, adjustment for dietary folate did not change the observed risk estimates in this and our previous analysis. [16] In this analysis, we have also adjusted for other potential risk factors that were adjusted for in other studies but not included in our previous analysis (dietary calcium intake, smoking intensity, menopausal status, ever use of oral contraceptives, and ever use of menopausal hormones), and the strength of the observed associations remained significant.

The extent to which confounding variables inter-relate and influence the fibre-colorectal cancer relationship may vary between studies. These differences impact on study risk estimates and could explain some of the disparities in results. However, the magnitude of the risk estimate changes between the least adjusted and multivariable adjusted models in our analysis and the Pooling Project analysis are similar, therefore differences in adjustment strategies are unlikely to explain the difference in results. Although residual confounding cannot be discounted, interaction analyses and models with different levels of adjustment revealed limited evidence that our inverse associations were caused by this. We observed non-significant interactions for BMI, waist circumference, age at recruitment, smoking, educational level attained, physical activity level, and intakes of alcohol, red and processed meat, calcium, and folate.

Dietary measurement error could also account for the lack of associations observed in some studies. This may cause modest dietary associations to be attenuated towards the null. [35], [36] In our analysis the inverse association of total fibre intake and colorectal cancer was strengthened after regression calibration using an additional dietary assessment (24-hour dietary recall) collected from a sub-set of cohort participants. For proximal and distal colon cancers, the calibrated models may have been unstable due to the high number of covariates included in the models and the relatively small number of cases after stratification by study centre. However, this method has been shown to lessen the impact of measurement error associated with dietary questionnaires. [32], [36].

In our previous analyses, the inverse associations were not attributable to fibre from a particular source. [16] The statistically significant 11% decrease in colorectal cancer risk per 10 g/day of cereal fibre intake we observed with longer term follow-up is similar to the estimate reported in the recent WCRF/AICR continuous update project meta-analysis. [18] It has to be taken into account that cereals are the main source of dietary fibre in most populations in the EPIC study. [34] When we combined fibre from fruit and vegetable sources (resulting in a comparable intake range to fibre from cereals) we obtained similar inverse associations for colon cancer to those for fibre from cereals. However, fibre from cereals but not fruit and vegetables was associated with rectal cancer.

A limitation of our study is that diet was only assessed at baseline, and that any potential dietary changes during follow-up are unaccounted for. However, the consistency of the inverse association of fibre intake with colorectal cancer risk observed throughout the duration of follow-up indicates that regression dilution is unlikely to have impacted upon our results. Strengths of our study include its large-scale prospective design, the large number of colorectal cancer cases, the possibility of controlling for the main potential confounders, and the partial correction for the effect of dietary assessment measurement error through regression calibration.

In conclusion, after 11 years of follow-up, this analysis of EPIC data confirmed the inverse associations between dietary fibre intake and colorectal cancer. These results strengthen the evidence for the recommendation of increasing the consumption of fibre rich foods for colorectal cancer prevention. [37].

## Supporting Information

Figure S1 **Nonparametric regression curve for the association between dietary fibre intake and colorectal cancer risk.** Hazard ratios estimated using a Cox proportional hazards model, adjusted for total energy intake (continuous), body mass index (continuous), physical activity index (inactive, moderately inactive, moderately active, active, or missing), smoking status and intensity (never; current, 1–15 cigarettes per day; current, 16–25 cigarettes per day; current, 16+ cigarettes per day; former, quit ≤10 years; former, quit 11–20 years; former, quit 20+ years; current, pipe/cigar/occasional; current/former, missing; unknown), education status (none, primary school completed, technical/professional school, secondary school, longer education including university, or not specified), ever use of contraceptive pill (yes, no, or unknown), ever use of menopausal hormone therapy (yes, no, or unknown), menopausal status (premenopausal, postmenopausal, perimenopausal/unknown menopausal status, or surgical postmenopausal), and intakes of alcohol, folate, red and processed meat, and calcium (all continuous), and stratified by age (1-year categories), sex, and centre. Solid line indicates HR, and dash lines indicate 95% confidence intervals derived from restricted cubic spline regression, with knots placed at the medians of each quintile of the distribution of fibre intake.
(TIF)

Table S1 **Multivariable hazard ratios (95% confidence intervals) of colorectal cancer risk in women by cohort wide sex-specific total dietary fibre intake quintiles.**
(DOCX)

Table S2 **Multivariable hazard ratios (95% confidence intervals) of colorectal cancer risk in men by cohort wide sex-specific total dietary fibre intake quintiles.**
(DOCX)
