# Supplementary figures and images for: Dietary Fibre Intake and Risks of Cancers of the Colon and Rectum in the European Prospective Investigation into Cancer and Nutrition (EPIC)
Source: PLoS One. 2012 Jun 22;7(6):e39361. doi: 10.1371/journal.pone.0039361 (PMC3382210; doi:10.1371/journal.pone.0039361)

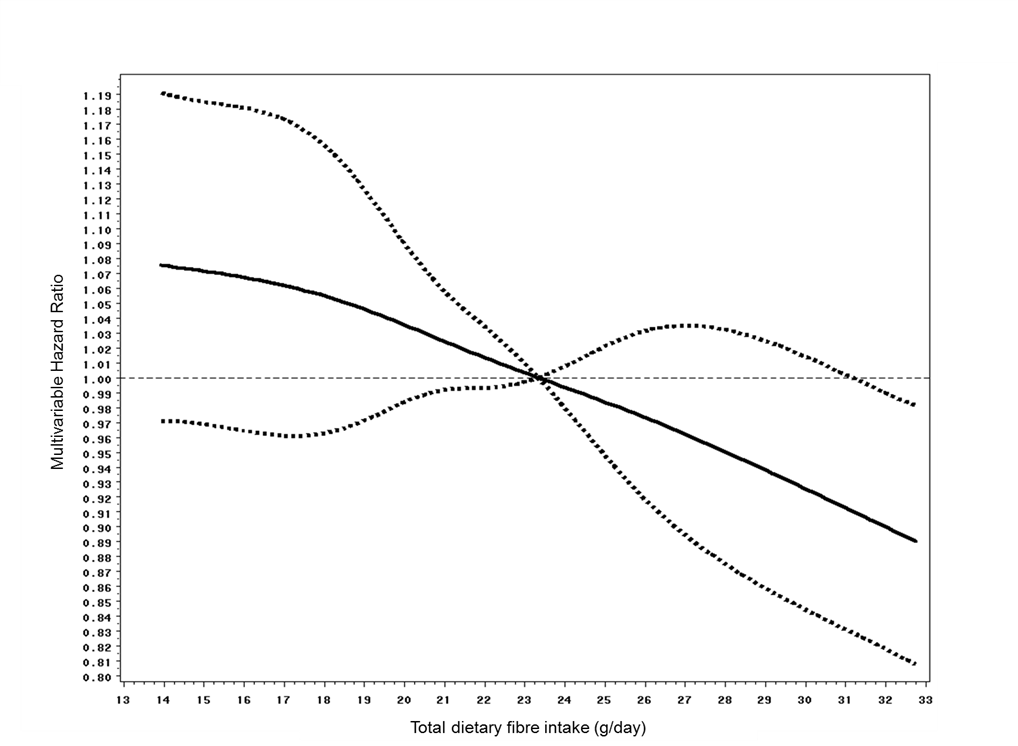

Supplement: Figure S1 — Nonparametric regression curve for the association between dietary fibre intake and colorectal cancer risk. Hazard ratios estimated using a Cox proportional hazards model, adjusted for total energy intake (continuous), body mass index (continuous), physical activity index (inactive, moderately inactive, moderately active, active, or missing), smoking status and intensity (never; current, 1–15 cigarettes per day; current, 16–25 cigarettes per day; current, 16+ cigarettes per day; former, quit ≤10 years; former, quit 11–20 years; former, quit 20+ years; current, pipe/cigar/occasional; current/former, missing; unknown), education status (none, primary school completed, technical/professional school, secondary school, longer education including university, or not specified), ever use of contraceptive pill (yes, no, or unknown), ever use of menopausal hormone therapy (yes, no, or unknown), menopausal status (premenopausal, postmenopausal, perimenopausal/unknown menopausal status, or surgical postmenopausal), and intakes of alcohol, folate, red and processed meat, and calcium (all continuous), and stratified by age (1-year categories), sex, and centre. Solid line indicates HR, and dash lines indicate 95% confidence intervals derived from restricted cubic spline regression, with knots placed at the medians of each quintile of the distribution of fibre intake. (TIF) [file pone.0039361.s001.tif]
