# Supplementary material for: Dietary Fibre Intake and Risks of Cancers of the Colon and Rectum in the European Prospective Investigation into Cancer and Nutrition (EPIC)
Source: PLoS One. 2012 Jun 22;7(6):e39361. doi: 10.1371/journal.pone.0039361 (PMC3382210; doi:10.1371/journal.pone.0039361)
Supplement: Table S1 — Multivariable hazard ratios (95% confidence intervals) of colorectal cancer risk in women by cohort wide sex-specific total dietary fibre intake quintiles. (DOCX) [file pone.0039361.s002.docx]

|  | |  | **Quintile of total fibre intake** | |  |  |  |  |  |
| --- | --- | --- | --- | --- | --- | --- | --- | --- | --- |
| **Fibre intake range (g/day)** | | | **1** | **2** | **3** | **4** | **5** |  | **HR (95% CI) per** |
| **Women** | | | **<16.1** | **16.1 - 19.5** | **19.6 - 22.9** | **23.0 - 27.4** | **≥27.5** | ***P*-*trend*** | **10 g/day increase *** |
| **Colorectum** | | |  |  |  |  |  |  |  |
|  | *N* cases | | 525 | 542 | 549 | 500 | 497 |  |  |
|  | Basic † | | 1.00 | 1.00 (0.88-1.13) | 0.98 (0.86-1.11) | 0.87 (0.76-0.99) | 0.83 (0.71-0.97) | 0.005 |  |
|  | Multivariable ‡ | | 1.00 | 1.02 (0.90-1.15) | 1.01 (0.88-1.15) | 0.90 (0.77-1.05) | 0.87 (0.72-1.05) | 0.063 | 0.89 (0.80-0.97) |
| **Colon** | | |  |  |  |  |  |  |  |
|  | *N* cases | | 357 | 371 | 355 | 351 | 318 |  |  |
|  | Basic † | | 1.00 | 1.01 (0.87-1.18) | 0.93 (0.80-1.09) | 0.91 (0.77-1.07) | 0.79 (0.65-0.96) | 0.007 |  |
|  | Multivariable ‡ | | 1.00 | 1.03 (0.88-1.20) | 0.96 (0.81-1.13) | 0.93 (0.78-1.12) | 0.82 (0.65-1.03) | 0.064 | 0.87 (0.78-0.98) |
| **Colon - proximal** | | |  |  |  |  |  |  |  |
|  | *N* cases | | 162 | 171 | 178 | 157 | 152 |  |  |
|  | Basic † | | 1.00 | 1.04 (0.83-1.30) | 1.05 (0.83-1.32) | 0.92 (0.72-1.17) | 0.86 (0.65-1.14) | 0.18 |  |
|  | Multivariable ‡ | | 1.00 | 1.06 (0.85-1.33) | 1.08 (0.85-1.37) | 0.95 (0.72-1.24) | 0.88 (0.63-1.23) | 0.32 | 0.87 (0.74-1.03) |
| **Colon - distal** | | |  |  |  |  |  |  |  |
|  | *N* cases | | 156 | 164 | 145 | 153 | 128 |  |  |
|  | Basic † | | 1.00 | 1.02 (0.82-1.28) | 0.88 (0.69-1.12) | 0.92 (0.71-1.19) | 0.77 (0.57-1.03) | 0.055 |  |
|  | Multivariable ‡ | | 1.00 | 1.02 (0.81-1.28) | 0.88 (0.68-1.14) | 0.92 (0.69-1.22) | 0.76 (0.53-1.09) | 0.12 | 0.87 (0.73-1.05) |
| **Rectum** | | |  |  |  |  |  |  |  |
|  | *N* cases | | 168 | 171 | 194 | 149 | 179 |  |  |
|  | Basic † | | 1.00 | 0.97 (0.78-1.21) | 1.07 (0.86-1.34) | 0.80 (0.62-1.02) | 0.93 (0.71-1.21) | 0.32 |  |
|  | Multivariable ‡ | | 1.00 | 1.00 (0.80-1.24) | 1.11 (0.88-1.40) | 0.84 (0.64-1.09) | 0.99 (0.72-1.35) | 0.63 | 0.92 (0.79-1.08) |

**Table S1.** Multivariable hazard ratios (95% confidence intervals) of colorectal cancer risk in women by cohort wide sex-specific total dietary fibre intake quintiles.

† Basic model - Cox regression using total energy intake (continuous), and stratified by age (1-year categories), and centre.
‡ Multivariable model - Cox regression using total energy intake (continuous), body mass index (continuous), physical activity index (inactive, moderately inactive, moderately active, active, or missing), smoking status and intensity (never; current , 1-15 cigarettes per day; current, 16-25 cigarettes per day; current, 16+ cigarettes per day; former, quit ≤10 years; former, quit 11-20 years; former, quit 20+ years; current, pipe/cigar/occasional; current/former, missing; unknown), education status (none, primary school completed, technical/professional school, secondary school, longer education including university, or not specified), ever use of contraceptive pill (yes, no, or unknown), ever use of menopausal hormone therapy (yes, no, or unknown), menopausal status (premenopausal, postmenopausal, perimenopausal/unknown menopausal status, or surgical postmenopausal), and intakes of alcohol, folate, red and processed meat, and calcium (all continuous), and stratified by age (1-year categories), and centre.
* Uncalibrated model shown.
