# Supplementary material for: Dietary Fibre Intake and Risks of Cancers of the Colon and Rectum in the European Prospective Investigation into Cancer and Nutrition (EPIC)
Source: PLoS One. 2012 Jun 22;7(6):e39361. doi: 10.1371/journal.pone.0039361 (PMC3382210; doi:10.1371/journal.pone.0039361)
Supplement: Table S2 — Multivariable hazard ratios (95% confidence intervals) of colorectal cancer risk in men by cohort wide sex-specific total dietary fibre intake quintiles. (DOCX) [file pone.0039361.s003.docx]

**Table S2.** Multivariable hazard ratios (95% confidence intervals) of colorectal cancer risk in men by cohort wide sex-specific total dietary fibre intake quintiles.

|  |  | **Quintile of total fibre intake** | |  |  |  |  |  |
| --- | --- | --- | --- | --- | --- | --- | --- | --- |
| **Fibre intake range (g/day)** | | **1** | **2** | **3** | **4** | **5** |  | **HR (95% CI) per** |
| **Men** | | **<17.3** | **17.3 - 21.3** | **21.4 - 25.3** | **25.4 - 30.5** | **≥30.6** | ***P*-*trend*** | **10 g/day increase *** |
| **Colorectum** | |  |  |  |  |  |  |  |
|  | *N* cases | 435 | 411 | 386 | 362 | 310 |  |  |
|  | Basic † | 1.00 | 0.92 (0.80-1.06) | 0.86 (0.74-1.00) | 0.80 (0.68-0.94) | 0.69 (0.58-0.83) | <0.001 |  |
|  | Multivariable ‡ | 1.00 | 0.97 (0.84-1.12) | 0.94 (0.80-1.10) | 0.90 (0.76-1.08) | 0.83 (0.66-1.03) | 0.08 | 0.91 (0.83-1.01) |
| **Colon** | |  |  |  |  |  |  |  |
|  | *N* cases | 265 | 246 | 230 | 192 | 184 |  |  |
|  | Basic † | 1.00 | 0.91 (0.76-1.09) | 0.86 (0.71-1.04) | 0.71 (0.57-0.87) | 0.69 (0.54-0.87) | <0.001 |  |
|  | Multivariable ‡ | 1.00 | 0.95 (0.79-1.14) | 0.91 (0.75-1.12) | 0.77 (0.61-0.97) | 0.77 (0.58-1.03) | 0.039 | 0.91 (0.80-1.03) |
| **Colon - proximal** | |  |  |  |  |  |  |  |
|  | *N* cases | 109 | 99 | 98 | 85 | 87 |  |  |
|  | Basic † | 1.00 | 0.90 (0.68-1.19) | 0.92 (0.69-1.23) | 0.81 (0.59-1.11) | 0.83 (0.58-1.19) | 0.27 |  |
|  | Multivariable ‡ | 1.00 | 0.93 (0.70-1.24) | 0.98 (0.72-1.34) | 0.88 (0.62-1.25) | 0.95 (0.62-1.47) | 0.77 | 0.96 (0.79-1.16) |
| **Colon - distal** | |  |  |  |  |  |  |  |
|  | *N* cases | 135 | 107 | 114 | 85 | 79 |  |  |
|  | Basic † | 1.00 | 0.78 (0.60-1.02) | 0.83 (0.64-1.09) | 0.61 (0.45-0.83) | 0.58 (0.41-0.81) | <0.001 |  |
|  | Multivariable ‡ | 1.00 | 0.83 (0.64-1.09) | 0.91 (0.68-1.21) | 0.69 (0.49-0.97) | 0.68 (0.44-1.05) | 0.061 | 0.89 (0.73-1.08) |
| **Rectum** | |  |  |  |  |  |  |  |
|  | *N* cases | 170 | 165 | 156 | 170 | 126 |  |  |
|  | Basic † | 1.00 | 0.94 (0.75-1.17) | 0.87 (0.69-1.09) | 0.94 (0.74-1.19) | 0.71 (0.53-0.94) | 0.029 |  |
|  | Multivariable ‡ | 1.00 | 1.01 (0.81-1.27) | 0.98 (0.76-1.25) | 1.11 (0.85-1.46) | 0.91 (0.64-1.29) | 0.79 | 0.92 (0.79-1.08) |

† Basic model - Cox regression using total energy intake (continuous), and stratified by age (1-year categories), and centre.
‡ Multivariable model - Cox regression using total energy intake (continuous), body mass index (continuous), physical activity index (inactive, moderately inactive, moderately active, active, or missing), smoking status and intensity (never; current , 1-15 cigarettes per day; current, 16-25 cigarettes per day; current, 16+ cigarettes per day; former, quit ≤10 years; former, quit 11-20 years; former, quit 20+ years; current, pipe/cigar/occasional; current/former, missing; unknown), education status (none, primary school completed, technical/professional school, secondary school, longer education including university, or not specified), and intakes of alcohol, folate, red and processed meat, and calcium (all continuous), and stratified by age (1-year categories), and centre.
* Uncalibrated model shown
